# Co-emergence and Collapse: The Mesoscopic Approach for Conceptualizing and Investigating the Functional Integration of Organisms

**DOI:** 10.3389/fphys.2019.00924

**Published:** 2019-07-26

**Authors:** Mariano Bizzarri, Alessandro Giuliani, Andrea Pensotti, Emanuele Ratti, Marta Bertolaso

**Affiliations:** ^1^Systems Biology Group Lab, Department of Experimental Medicine, Sapienza University of Rome, Rome, Italy; ^2^Department of Environment and Health, Istituto Superiore di Sanità, Rome, Italy; ^3^FAST, University Campus Bio-Medico, Rome, Italy; ^4^Reilly Center for Science, Technology, and Values, University of Notre Dame, Notre Dame, IN, United States

**Keywords:** living dynamics, systems thinking, mesoscopic way, data emergence, micro-environment, physical constraints, relational ontology, biological relationships

## Abstract

The fall of reductionist approaches to explanation leaves biology with an unescapable challenge: how to decipher complex systems. This entails a number of very critical questions, the most basic ones being: “What do we mean by ‘complex’?” and “What is the system we should look for?” In complex systems, constraints belong to a higher level that the molecular one and their effect reduces and constrains the manifold of the accessible internal states of the system itself. Function is related but not deterministically imposed by the underlying structure. It is quite unlikely that such kind of complexity could be grasped by current approaches focusing on a single organization scale. The natural co-emergence of systems, parts and properties can be adopted as a hypothesis-free conceptual framework to understand functional integration of organisms, including their hierarchical or multilevel patterns, and including the way scientific practice proceeds in approaching such complexity. External, “driving” factors – order parameters and control parameters provided by the surrounding microenvironment – are always required to “push” the components’ fate into well-defined developmental directions. In the negative, we see that in pathological processes such as cancer, organizational fluidity, collapse of levels and dynamic heterogeneity make it hard to even find a level of observation for a stable explanandum to persist in scientific practice. Parts and the system both lose their properties once the system is destabilized. The mesoscopic approach is our proposal to conceptualizing, investigating and explaining in biology. “Mesoscopic way of thinking” is increasingly popular in the epistemology of biology and corresponds to looking for an explanation (and possibly a prediction) where “non-trivial determinism is maximal”: the “most microscopic” level of organization is not necessarily the place where “the most relevant facts do happen.” A fundamental re-thinking of the concept of causality is also due for order parameters to be carefully and correctly identified. In the biological realm, entities have relational properties only, as they depend ontologically on the context they happen to be in. The basic idea of a relational ontology is that, in our inventory of the world, relations are somehow prior to the relata (i.e., entities).

## Complexity

Complexity as a concept emerged as a necessary stance – from both an epistemological and an ontological point of view – once the reductionist approach established since Descartes’s time proved to be inadequate in explaining a number of relevant phenomena. Such inadequacy is particularly evident in biology and resonates with the concept of “unfathomable complexity,” proposed by Elsasser (1987). This notion of complexity has to do with the impossibility to devise a series of experiments to clarify the way in which all properties of an organism can be *reduced* to consequences of molecular structure and dynamics, the latter being controlled and fully determined by the laws of physics. The fall of reductionist approaches to explanation leaves biology with an unescapable challenge: how to decipher complex systems. This apparently simple association of words entails a number of very critical questions, the most basic ones being: “*What do we mean by ‘complex’*?” and “*What is the system we should look for*?”

Besides the existence of several excellent operational definitions of complexity elaborating on different notions of the amount of correlation among parts of a system (see for example: 28, 30, 31, 32), herewith we sketch three relevant “signatures” of complex systems:

1. A complex system includes a vast number of components (nodes), linked each other through dynamic relationships (links), enabling its representation in terms of a correlation network.
2. Such a network (independently of the chosen correlation metrics) exhibits a hierarchical structure, allowing the system to have meaningful dynamics at different spatial and temporal scales.
3. The spatial and temporal relationships among the different elements are subdued to a non-linear dynamics giving rise to both memory (hysteresis) and multi-stability (different equilibrium states) effects.

Thereby, describing the evolving system in both space and time is not trivial, as different functional states of the system can be supported by the same underlying structure. Indeed, a complex system can occupy different attractors along the paths of a hypothetical landscape, as suggested by C.H. Waddington in the 1950s. According to the topological architecture of such landscape, the system displays various degrees of robustness, i.e., resilience in respect to internal/external perturbations. As sharply observed by Elsasser (1987), “If we accept the concept of an organism as just stated, we can say that biology is a non-Cartesian science.”

To be “non-Cartesian,” in this context, means simply that the system looks different at different magnification scales and no privileged (and context independent) explanation layer exists.

Therefore, it is quite unlikely that such kind of complexity could be grasped by current approaches focusing on a single organization scale: complexity does not depend either on the number of genes (indeed, humans show lower values in respect to even evolutionary lowest living individuals, as strawberry!), or on their connections [the so-called Gene Regulatory Network (GRN)]. As an example, it was posited that extensive search for genome structures among animals would have come to identify those genes that actually hold the key to humanness. Yet, the result of such an effort showed that humans and chimps are basically isogenic; no specific human genes responsible for our human properties could be identified (The Chimpanzee, Sequencing, and Analysis Consortium., 2005). Similar problems were present in the “gene centric” explanations of complex human traits and diseases. As aptly remarked, “although some niche applications have been found for precision medicine, and gene therapy is now becoming a reality for a few rare diseases, the effects on public health are minuscule while the costs are astronomical” (Joyner and Paneth, 2019).

Classical molecular studies focused on the dynamics of single molecular components conceived as “drivers” of the biological process. A major drawback of such approach that it is unable in explaining the emergence of complex patterns. The emergence of a pattern (i.e., of a relatively stable configuration of n elements being them gene expression levels or amino-acid residue positions in 3D space) has to do with the energy minimization on the entire n-dimensional space and cannot be traced back to separate “optima” for each element. The among elements correlation and the presence of environmental constraints drastically “restrict” the number of patterns that the system at hand can really assume (Kitano, 2002; Chuang et al., 2010; Ma’ayan, 2011).

A case in point is provided by microgravity conditioning of living cells, where the “removal” of the gravity constraints enable the system to freely explore new – previously “inaccessible” – phenotypic attractors, by splitting a previous homogeneous population into two – morphologically and functionally – different clusters (Masiello et al., 2014). This phenomenon cannot be understood by investigating the “molecular dynamics” of the involved components, and it should be viewed as a true “emergent” property of the system, triggered by an environmental, physical cue. Constraints belong to a higher level that the molecular one and the aforementioned effect reduces and constrains the manifold of the accessible internal states of the system itself. Conclusively, the form a molecule/a cell assume cannot be linearly derived (“reduced”) only from the physical laws governing combinatorics.

This is a crucial issue separating the operational measures of complexity (mainly applied to alphabetical or numerical strings) from “semantic sensitive” functionally oriented definitions: “sequence complexity” does not mirror “structural complexity” of the organism that the sequence gives rise to. This statement applies (among the others) to Kolmogorov complexity measure, as this index is a degree of regularity (correlation in time), rather than of semantic-functional complexity (a random sequence is accorded maximum Kolmogorov complexity, yet, a biologist could hardly be interested with, given that random sequences do not give rise to organisms) (Adami, 2002). Along the suggestions fostered by Kolmogorov-dependent definition of complexity, several attempts have been made to correlate (and even to equate) complexity to entropy measures. However, entropy in dissipative systems does not increase, but eventually decreases just as negative entropy corresponds to (relative) order, certainty, and organization (Mikhailovsky and LevichEntropy, 2015). This implies that Kolmogorov measure proceeds likely in the opposite way from classical, entropy-based, order parameters.

This conundrum lies on the deceptive assumption that equalize “order” and “complexity.” Yet, complex system are neither fully disorganized (like a gas), nor steadily ordered (like a crystal). In complex system order values are changing over time, showing dramatic fluctuation at points where the system undergoes critical transitions, leading to the emergence of new configurations (“phenotypes,” in the biological parlance) in which order values (i.e., negentropy) is frequently uncoupled from Kolmogorov-based complexity parameters. As a result, changes in entropy may likely reflect true differences in system’s order, but not necessarily in complexity. Moreover, in biological systems, complexity cannot be computed based on the number of functions they fulfill. Differentiated cells lose several capabilities when compared to their progenitor stem cells, yet it would be paradoxical to affirm that such a highly structured cell like a neuron is deprived of complexity. To make a long story short, we can state “In biology function is related but not deterministically imposed by the underlying structure.”

## Co-Emergence and Collapse

Co-emergence of system, parts and properties can be adopted as a conceptual framework to understand functional integration of organisms, including their hierarchical or multilevel patterns, and including the way scientific practice proceeds in approaching such complexity (Bizzarri et al., 2013; Bertolaso, 2016).

Complex systems (paradigmatically those entailed by living beings) show emergent properties, which likely arise from the non-linear dynamics of the relationships established among the entities (molecular components) that make up the system. This statement implies some non-trivial consequences, at both the epistemological and ontological level.

First, as forecasted by Whitehead, biological phenomena consists of processes rather than material objects, and that processes are best defined by their relations with other processes, thus undermining the common shared belief on “bits of matter” – like genes or other molecular units – that exist and function independently of one another (Whitehead, 1967). Accordingly, molecular components are nothing more and nothing less than the sum of their relations to other entities, “its synthesis of and reaction to the world around it” (Whitehead, 1985). As “all things flow” – a philosophical stance that can be traced back to Heraclitus and that has been adopted recently in contemporary philosophy of science (Duprè and Nicholson, 2018) – it is mandatory to shift our focus from “molecules” to “relationships.”

A phenomenon can in principle be studied at different levels (sub-atomic, atomic, molecular, cell, etc.), but we are mostly interested in “effective” relationships, i.e., those relations triggering “emerging properties” of the system at a higher organization layer. Effective relations live at the “mesoscopic level,” as pinpointed by Laughlin and Pines (2000). This is the level where “order” can be fruitfully found, as only systems behave in a coherent fashion (even gene activity is subjected to intrinsic stochastic fluctuation (Elowitz et al., 2002). Indeed, while at the microscopic level, objects and their relationships are affected by fluctuations around the average – due to both environmental and intrinsic stochasticity – at the mesoscopic level stochastic fluctuations turn into ordered behavior, thus allowing order to emerge. A useful architectural analog of the mesoscopic level are the arches of a gothic cathedral: the arch occupies the intermediate layer between the brick and the entire building and represents the optimal level where to study the forces responsible for the stability of the cathedral as a whole.

The aforementioned reflections push to reconsider the current epistemological approach, concepts borrowed from classical mechanics – like those referring to determinism in biological reactions and causality – do not hold the same meaning when we are referring to the mesoscopic level and need to be re-framed accordingly. Going back to the architectural metaphor the statement “The arch generates (is the ‘cause’ of) the cathedral structure” is devoid of any sense, and must be substituted by “The presence of a given push-pull momenta configuration correspondent to arches network drives the entire cathedral toward an ‘allowed’ (stable) global structure.” Deterministic (if-then) causation is substituted by the drastic restriction of “allowed solutions” stemming from the underlying mesoscopic configuration.

Co-emergence of systems, parts and properties are a natural (and hypothesis free) consequence that can be adopted as a conceptual framework to understand functional integration of organisms, including their hierarchical or multilevel patterns, and including the way scientific practice proceeds in approaching such complexity (Bizzarri et al., 2013). This can be crucially important in such pathological processes such as cancer, where organizational fluidity, collapse of levels and dynamic heterogeneity, make it hard to decide *a priori* a specific level of observation for a stable *explanandum* (what must be explained) to persist in scientific practice. Parts and the system both lose their properties once the system is destabilized. In the negative, we see that in pathological processes such as cancer, organizational fluidity, collapse of levels and dynamic heterogeneity make it hard to even find a level of observation for a stable *explanandum* to persist in scientific practice. Parts and the system both lose their properties once the system is destabilized.

This specifically holds true in carcinogenesis, were some key aspects of tumor development (metastasis, phenotypic transition, growth or dormancy) emerge from the non-linear dynamics of the interactions between cells and their microenvironment (Bizzarri and Cucina, 2014). Changes in the tissue microenvironment act as stress factors on cells causing a range of adaptive responses within the reaction norm of the genome that may at some stage also include higher mutation rates. Tissue stress factors can be changes in the ECM composition (Extra-Cellular Matrix) caused by exposure to some carcinogen (and most carcinogens are not primary mutagens), changes in mechanical tissue forces after trauma, surgery and wound healing, or a change in fundamental signaling interactions between groups of cells due to changes in pH, Oxygen balance, and metabolic conditions which are all progressively changing during the course of a tumor’s evolution. More generally, the life history of the biological entity intrinsically depends on a constitutive and continuous orientation of the parts among themselves and depending on the contextual signals. The asymmetry so generated is vital in the sense that it guarantees the adequate growth of the organism, as the effects of changes in cell or tissue shape seem to show. This unity of action admits degrees, and parts-whole relationships – as long as they hold – are to be explained through a specific kind of regulation. The biology of cancer shows that the stability of constitutive elements depends on the organization, and that there is a source of regulation in the biological context: cells change their behavior depending on their functional integration in the tissue; alteration in cell communication in turn alters gene expression, and the loss of integration of cells within a functional tissue leads to genetic instability and apoptosis.

The collapse of levels, as characterized in cancer, results from the loss of the general functional integration of a biological entity. It is here that the “mesoscopic level,” as pinpointed by Laughlin, is substantiated.

## The Mesoscopic Approach

The described situation highlights the importance of identifying an explanatory level that is adequate to the observed phenomenon, by finding relational structures that are able to relate microscopic elements and macroscopic phenomena and parameters. “Mesoscopic way of thinking” is an increasingly popular statement in the epistemology of biology (Noble, 2006; Bertolaso, 2009; Bertolaso, 2016) and corresponds to looking for an explanation (and possibly a prediction) based on such kind of description. “Mesoscopic” is a term that originates in physics and engineering, and very frequently adopted in Ecology, perhaps the most epistemologically conscious branch of biology (Hastings et al., 2011).

Ecologists have long recognized that the “most microscopic” level of organization is not necessarily the place where “the most relevant facts do happen.” On the contrary, the most fruitful scale of investigation (most of the times) is where “non-trivial determinism is maximal” (Pascual and Levine, 1999). That is to say, the scale more “rich” in meaningful correlations between features pertinent to micro- and macro- scale or, to use an ecological term, the mesoscopic realm. Non-trivial determinism can be, in fact, defined in terms of prediction error as:

$$\text{Prediction}\;{\text{r}}^{2}=1-{\text{E}}^{2}/{\text{S}}^{2}$$

In the above formula, *E* is the mean prediction error and *S* the standard deviation. In the case of a simple linear regression in which a dependent variable *Y* must be predicted by an independent variable *X*, non-trivial determinism is nothing else than the usual squared Pearson correlation between the two *X* and *Y* variables. The formula can be extended to any other situation in which we wish to predict a system feature *Y* located at a hierarchically higher layer with respect to *X*, moreover both *X* and *Y* do not need to represent single variables but any suitable set of information at any definition scale. Consequently, in the “many *Y*”/“many *X*” case, non-trivial determinism corresponds to the first canonical coefficient (Härdle and Simar, 2007) while in the case of a binary diagnosis it equates to the area below the ROC curve (Heagerty and Zheng, 2005).

The mesoscopic way of reasoning closely resembles the “middle-out” paradigm set forth by Laughlin et al. (2000) as the next frontier of basic science – of chemistry, quantum physics for coherent dynamic behaviors (Bertolaso et al., 2015), and, more recently, of network-based approaches in biology (Giuliani et al., 2014). Complex networks (whichever level of biological organization they belong to) allow for a natural convergence between top-down and bottom-up approaches for the simple fact the computation of network invariants encompasses the simultaneous consideration of microscopic (single node), mesoscopic (cluster of nodes) and macroscopic (entire network) features (Csermely et al., 2013). This allows the environmental effects at different levels of definition to be tracked (Masiello et al., 2018).

This implies that statements like “*Drug A provokes a drastic increase of average shortest path of protein contact network*” or “*Cancer provokes a decrease in modularity of gene regulation network*” should be accepted as meaningful explanations without the need to go in depth into specific amino-acid residues or genes. When looking at networks, functional properties of a node are inferred from its topological role in the network. However, without the right choice of mesosystems and the appropriate estimation of local/global constraints, the problem of finding elements that are causally specific with respect to the initial *explanandum* will not be solved, no matter which mechanisms at the lowest (e.g., molecular) level are found. On the other hand, empirical topology/function rules are discovered at different degrees of generalization by moving from a population of heterogeneous biological networks to a single wiring architecture (Kohestani et al., 2018).

It is worth noting the same concepts (here mainly linked to spatial organization) apply to temporal structures. Recurrence Quantification Analysis (RQA) (Marwan et al., 2007) is a non-linear signal analysis technique focusing on the search of “non-trivial determinism” that here takes the form of “sojourn points,” i.e., areas of the phase space that are visited by different system trajectories corresponding to “stable configurations” of the temporal organization.

The onset of such “deterministic islands” in a time course is instrumental to detecting emerging properties of the system as a whole: e.g., the onset of “fatigue” (a global system feature), corresponds to the observation of a drastic increase in determinism of EMG time series (a mesoscopic (muscle fibers) level feature) (Liu et al., 2004).

### Conceptualizing the Mesoscopic Way

The mesosystem is an identified system that exhibits regularities where not only the peculiar relations among the parts, but also the properties of the parts themselves and their reciprocal interactions, emerge (Laughlin et al., 2000). Laughlin and Pines (2000) state: “*The emergent physical phenomena regulated by higher organizing principles have a property, namely their insensitivity to microscopic details that is directly relevant to the broad question of what is knowable in the deepest sense of the term*.”

Insensitivity to microscopic details is the core of the middle-way and stems from the possibility to establish a “network thermodynamics” (Mikulecky, 2001), building upon the existence of laws of nature only dependent on the wiring architecture of a system that co-exist (but do not interfere) with the laws typical to the material the elements are made of. That is why an electrical circuit can simulate a mechanical one and, after all, why we do not need to enter into the details of electronic properties of single constituent atoms to get onto enzyme kinetics.

The most basic mathematical construct of the mesoscopic approach is the “complex network” wherein a set of nodes are linked to each other by edges. Nodes can be any relevant element of the system at hand (genes, proteins, amino-acid residues, neurons) and edges any connection between them (correlation coefficients, physical interactions, spatial proximities, simultaneous firing). Network invariants are nothing else than descriptors of the corresponding wiring architecture such as:

1. “degree”: how many links are attached to a given node, which is a local descriptor,
2. “average shortest path,” corresponding to the average length of minimal paths connecting all the node pairs, which is a mesoscopic feature.
3. “connectivity” that is the density of links, which in turn is a global property.

**FIGURE 1:**
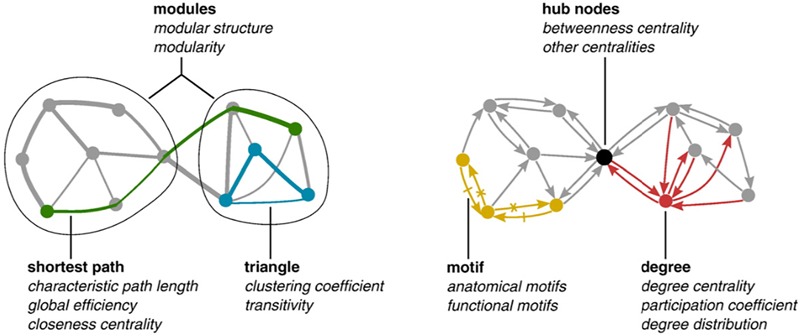
Modules correspond to subset of nodes having much more links among them than with other nodes of the network. Measures of centrality (closeness, betweenness) describe nodes in terms of the number of shortest paths traversing them. Shortest path is the characteristic metrics for networks: they correspond to the shortest distances (in terms of number of nodes/links to be traversed) for linking pairs of nodes.

Figure 1 depicts some of these descriptors.

It is immediate to note that the values taken by the above descriptors depend (and influence) all the different organization layers: thus a node with a high degree (microscopic level) will be traversed by many shortest paths (mesoscopic level) that in turn will influence general network connectivity (macroscopic level).

In operational terms, shortest paths spanning protein contact networks (those networks whose nodes are amino-acid residues and whose edges correspond to residues set apart along the sequence and put in contact by protein folding) correspond to the “fast lanes” along which allosteric signals travel. This creates an immediate link between a global functional property of the protein molecule (allostery) and the mesoscopic level (shortest paths) strictly resembling the architectural metaphor we introduced in section “Complexity” (arches = shortest paths, gravitational forces = allosteric signal) (Di Paola and Giuliani, 2015).

It is worth noting that this approach is by no means limited to complex networks: any meaningful representation of the correlation structure of a system can extract relevant “mesoscopic principles of organization.” This is the case of a time-honored technique, principal component analysis (Pearson, 1901; Giuliani, 2017), which allows for an immediate quantitative appreciation of the degree of order and organization of a system (Soofi, 1994).

Success of the mesoscopic approach strictly depends upon choice of the correct level (and consequently the correct observables) to be investigated. Choice of the “preferable” level is dictated by the function-phenomenon we are focusing on. It is at this level – the mesoscopic one – that microscopic elements are organized in a coherent manner so to produce macroscopic features. The mesoscopic approach strives to “capture” the self-organizing process, which in turn will lead to the emergence of specific system properties.

That approach implies we should put any singularity (i.e., discrete change in a unitary molecular element) in dynamic correlation with the context it belongs to. Function and meaning of each molecular change emerges thereby within such a context, having no “ontological” meaning *per se*. This aspect is specifically epitomized when looking at the role played by those genes that can exert their effects in opposite ways, depending on the time/context in which they are activated. Conclusively, these findings imply the necessity to take into consideration, even when we concentrate on a single element (e.g., the lethal character of a specific mutation), the general functional frame in which the element is inserted.

## Investigating the Mesoscopic Way

Experimental practices both clarify the notions related to the mesoscopic approach and demonstrate its consistency and usefulness. In the previous section, we introduced the concept of non-trivial determinism as an operational guide for setting the optimal scale definition. The Pearson correlation can be substituted in the formula by any suitable correlation metrics as connectivity or average shortest path in the case of networks, amount of determinism in the case of recurrence analyses (Marwan et al., 2007). It is worth noting already that such statistical methodology allows for a quantitative check of the heuristic power of a given temporal or spatial scale of organization in terms of maximization of “non-trivial determinism” (Pascual and Levine, 1999).

**FIGURE 2:**
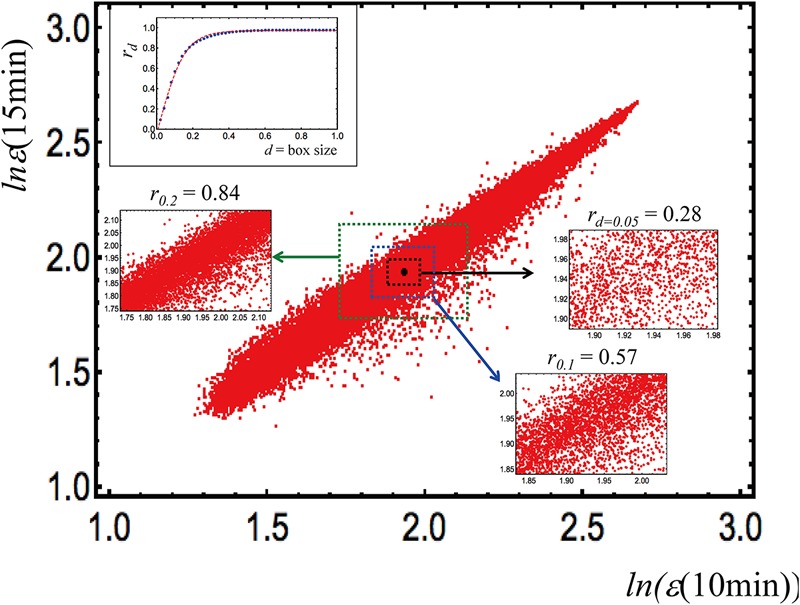
In order to visualize the ability of this approach to identify the optimal scale of analysis, we report in this figure, the bi-dimensional plot having as axes two independent MCF7 (a breast cancer derived cell line) samples (data obtained from Tsuchiya et al., 2016). The points of the graph (around 23000, expression values in logarithm units) are the single gene expression values, the *d*-value corresponds to the range (box size) of variation, inside which the correlation (Pearson coefficient, *r*) is computed.

In order to visualize the ability of this approach to identify the optimal scale of analysis, we report in Figure 2 the bi-dimensional plot having as axes two independent MCF7 (a breast cancer derived cell line) samples. The points of the graph (around 23000, expression values in logarithm units) are the single gene expression values, the *d*-value corresponds to the range (box size) of variation, inside which the correlation (Pearson coefficient, *r*) is computed.

The correlation computed overall is near to unity (*r* = 0.98), and declines at decreasing range of variation; the inset on the top left corner of the figure shows that a correlation plateau is reached at *d* = 0.45. This remark outlines how correlation values are tightly dependent on the observation scale: in this case, the optimal scale is where correlation between the two samples (correspondent to the existence of an ideal gene expression profile of such cell kind) reaches its maximum and corresponds to the correlation attainable with a random choice of 50 genes. This implies that the “minimal set” for making the “specific cell kind transcriptome signature” emerge is 50 genes, which in turn corresponds to a very important property of complex systems called “percolation” (Pike and Stanley, 1981) linked to the level of perturbation needed to provoke a transition in the system at hand (Tsuchiya et al., 2016).

In this peculiar case, the scale dependence of the correlation is instrumental to maintain both tissue functionality (the specialized physiological function asks for an invariant ideal pattern of gene expression) and the flexibility required to adapt to a changing microenvironment (the specific gene expression levels need a “free motion” range to cope with environment).

As the mesoscopic level is where organizational principles act on the elementary biological units that will become altered, or constrained, by both their mutual interaction and the interaction with the surrounding environment, constraints acting at the mesoscopic level can therefore shape the activity of single elements (proteins, genes, etc.), eventually driving them into different and even opposite functions. This explains why putative so-called oncogenes can act as tumor-suppressor genes (Lee et al., 1995; Johnson, 2000; Muñoz et al., 2008; Schneller et al., 2011; Wang et al., 2011), a well-known paradox that undermines the fundamentals of the Somatic Mutation Theory of carcinogenesis (Baker and Kramer, 2007; Bizzarri and Cucina, 2016).

## The Dynamics of Mesoscopic Parameters

As we are dealing with processes – no matter the number and the specific identities of the involved molecular components – in which mesoscopic properties of the system are thought to change in space and time, we should focus on the dynamics of mesoscopic parameters. A useful way is to adopt a landscape diagram in which system’s transitions from one state (attractor) to another is portrayed by means of calculating order and control parameters, by analogy with phase-space diagrams employed in physics and chemistry. Such a model is already currently in use, albeit framed within a reductionist stance. Indeed, the Waddington’s model usually adopted is featured by hills and valleys linked each other through branched pathways to portray the differentiation tree (Waddington, 1957). Both valleys and hills are determined by calculating values of state variables recognized by GRNs models, where the mean trajectory of observational data are obtained by numerically solving ordinary differential equations (ODE). This procedure usually leads to identifying several activation states, as featured by specific sub-sets of gene expression patterns (Wu et al., 2014). Based on this approach, a number of studies managed to demonstrate how intrinsic stochasticity in gene expression can activate a wide range of gene patterns, thus accounting for differences in phenotypes observed even among isogenic cell populations (Kupiec, 1983). The random pattern of gene expression produces probabilistic outcomes by activating switching mechanisms that select among alternative paths, ultimately leading an isogenic cell population to be partitioned into different phenotypes (McAdams and Arkin, 1997) because of the interplay between stochasticity of gene activity and non-linear dynamics of the transcriptional regulatory network (Huang, 2009). Current models posit that GRNs activity is modulated in a subtler fashion and results to be extremely sensitive to even small fluctuations occurring in the molecular dynamics in both the internal and the external microenvironment. Therefore, the GRN configuration can “capture” any kind of perturbation occurring in the system, although it is still unclear how GRNs activity could “sense” perturbations from the biophysical microenvironment. Moreover, GRNs sensitivity to external changes can hardly accommodate with one of the advantages displayed by complex systems, i.e., robustness (resilience to fluctuations).

## Relational Ontology for Biological Properties

Mesoscopic models are able to capture many characteristics of a system. But then, what kind of causality is able to support biological explanations? Perturbations coupled with intrinsic genomic stochasticity can both destabilize an attractor state thus resulting in different gene expression patterns that would support independent cell “identities.” The resulting presence of different transcriptional profiles at the bifurcation point is properly a “transient state” and it represents a raw substrate for cell fate switching, but in its own cannot decide about the fate the cell will choose and why a transition ends up into a unique phenotypic specification. By focusing only on the intrinsic dynamics of GRNs we could hardly find out why a cell population shall take a specific direction among many others. Namely, an unsolved problem is represented by what happens at the bifurcation points, where cell fate decisions take place (Moris et al., 2016). Is an external, “driving” factor required to “push” cell fate into one well-defined direction (Masiello et al., 2018)? Indeed, Waddington’s based diagram include both control parameters, mainly provided by the surrounding microenvironment, which mostly belong to the class of physical constraints, and order parameters. In fact, ordered phenomena might only arise from global cues and constraints that superimpose their driving effects upon the local, random dynamics of molecular agents (Bizzarri et al., 2013).

A preliminary re-thinking of the concept of causality is due to allow order parameters to be carefully and correctly identified. In classical physics, the individual item is subjected to rigorous causality recognition according to the paradigm of linearity briefly mentioned in section “Complexity”: effects are linearly transduced from causative factor(s) to end up into an effect in a deterministic fashion. In quantum mechanics, instead, such item is undetermined, whereas the average behavior of the including set (the “ensemble” in mathematical terms) can be defined in terms of statistical probability. That is to say, while in classical physics every individual object obeys causality, in quantum mechanics – also applying to complex systems – causality is meaningful only by considering an entire class of objects. In other words: causality applies to a class of entities, rather than to a single object. Therefore, the quantities/properties that are in common to all members of a class are the observables of the ensemble/system. As an example, consider cardiac rhythm: this is an outstanding (system) parameter, *per se* able to capture the functioning of the cardiac system and its possible pathological features. However, this rhythm does not pertain to any single myocardial cell or to single molecules either (Noble and Noble, 1984). It is a true system property, arising from the coordinated activity of the system, and it represent the parameter we must look at in order to grasp really the functioning of the cardiac muscle.

Noble (2017) emphasizes that the existence of system properties that constrain the behavior of lower-level entities is a case of downward causation – “the control of lower-level processes by higher-level processes” (p. 81). However, it is not exactly clear how this might be possible – if entities at lower levels have certain characteristics that make them behave in certain ways, how do we make sense of system constraints? By drawing on previous work (Bertolaso and Ratti, 2018), we propose to use a relational ontology to ground both the conceptual and explanatory aspects of this issue.

The basic idea of a relational ontology is that, in our inventory of the world, relations are somehow prior to the *relata* (i.e., entities). In biological terms, this would mean that the identity of biological entities should not be conceived in terms of their (internal) characteristics, but rather in terms of the relations they have with other entities – ultimately, in terms of the habitat they are embedded in. Therefore, understanding biology in terms of a relational ontology, means shifting the focus from the entities taken in isolation, to the historical context (bio-environment) of the entities themselves. Let us make these considerations more precise. An entity is a specific (i.e., biological) class of things that are subjected to the predication of properties. Simplifying, we can distinguish between intrinsic properties – properties that entities have in virtue of what they are (e.g., having a specific mass) – and relational properties – properties that entities have because of the way they interact with other entities (e.g., acidity). The idea behind a relational ontology is that this distinction is spurious. In fact, even properties that look like they are intrinsic, in fact they are relational. A case in point is the notion of gene (Boem et al., 2016). Genes can be specified in terms of intrinsic properties (i.e., the gene x is a specific sequence of nucleotides), while in other contexts such as in networks biology a specific gene is defined as a node within a network of interactions (Barabasi and Oltvai, 2004) defined by a set of descriptors related to its connectivity features (e.g., degree, clustering coefficient, etc.). However, as Bertolaso and Ratti (2018) say “the fact that a gene has a specific sequence, and the fact that this sequence has a certain causal role (i.e., being transcribed as a blueprint for a specific protein) strictly depend on the context where the gene happens to be. Therefore, even properties that seem prominently internal are somehow relational, i.e., they depend on the context.”

To specify this further, let us introduce the notion of “ontological dependency.” As Wolff clarifies, “[t]o say that A ontologically depends on B is to say that both A and B exist, but that B is in some sense ontologically and explanatorily prior to A (…) A exists (at least in part) because B exists” (p. 618). Therefore, in the biological realm entities have relational properties only, as they depend ontologically (in the sense just specified) on the context they happen to be in. This view can vindicate even more the relevance of the mesoscopic approach in contemporary biology. It also supports the thesis defended elsewhere (Plutynski and Bertolaso, 2018) concerning the explanatory import of systemic models when dealing with complex biological dynamics. They deal in fact with properties of signaling networks and concern general patterns of stability and instability in the dynamics of, for example, cancer progression. “This requires placing cell intrinsic mechanisms associated with cancer initiation and progression in a larger context, at a variety of temporal and spatial scales, from the cell-signaling networks active in wound healing to evolutionary and developmental history. In this way, they integrate top-down and bottom-up perspectives on the same phenomena” (ibidem). Robust feature of networks therefore are the target explananda of mesoscopic models.

## Conclusion

Organisms are complex entities co-emerging with their parts and properties. At the microscopic level, objects and their relationships are affected by fluctuations around the average – due to both environmental and intrinsic stochasticity – and then subject to quantum mechanics laws; at larger scales, stochastic fluctuations turn into ordered behavior, thus allowing order to emerge. The co-emergence of system, parts and properties – and the collapse thereof, as occurring in pathological processes such as cancer – can become a conceptual framework to understand the functional integration of organisms by adopting a mesoscopic approach, i.e., by focusing on the organization scale that – in network theory terms – maximizes the entity and number of correlations among the system’s elements. Experimental practices both clarify the notions related to the mesoscopic approach, and demonstrate its consistency and usefulness, as relevant results can be achieved only by means of right and sensible choice of mesosystem variables and the appropriate estimation of local/global constraints. A relational ontology is necessary to ground both the conceptual and explanatory aspects of the mesoscopic approach, driving a re-thinking of the concept of biological causality.

## Author Contributions

MBi and AP contributed to the scientific and biological sections. AG contributed to the bio-statistical aspects and statistics. MBe contributed to the overall structure of the manuscript, and philosophical and epistemological sections. ER contributed to the epistemological section.

## Conflict of Interest Statement

The authors declare that the research was conducted in the absence of any commercial or financial relationships that could be construed as a potential conflict of interest.
